# Identification of COVID-19 B-cell epitopes with phage-displayed peptide library

**DOI:** 10.1186/s12929-021-00740-8

**Published:** 2021-06-07

**Authors:** Jing-You Guo, I-Ju Liu, Hsiu-Ting Lin, Mei-Jung Wang, Yu-Ling Chang, Shin-Chang Lin, Mei-Ying Liao, Wei-Chia Hsu, Yi-Ling Lin, James C. Liao, Han-Chung Wu

**Affiliations:** 1 Institute of Biologic Chemistry, Academia Sinica, Taipei, Taiwan; 2 Institute of Cellular and Organismic Biology, Academia Sinica, No. 128, Academia Road, Section 2, Nangang, Taipei, 11529 Taiwan; 3 Institute of Biomedical Sciences, Academia Sinica, Taipei, Taiwan

**Keywords:** COVID-19, SARS-CoV-2, Phage-displayed peptides, B-cell epitope, Diagnostic tool, Serological detection

## Abstract

**Background:**

Coronavirus disease 19 (COVID-19) first appeared in the city of Wuhan, in the Hubei province of China. Since its emergence, the COVID-19-causing virus, SARS-CoV-2, has been rapidly transmitted around the globe, overwhelming the medical care systems in many countries and leading to more than 3.3 million deaths. Identification of immunological epitopes on the virus would be highly useful for the development of diagnostic tools and vaccines that will be critical to limiting further spread of COVID-19.

**Methods:**

To find disease-specific B-cell epitopes that correspond to or mimic natural epitopes, we used phage display technology to determine the targets of specific antibodies present in the sera of immune-responsive COVID-19 patients. Enzyme-linked immunosorbent assays were further applied to assess competitive antibody binding and serological detection. VaxiJen, BepiPred-2.0 and DiscoTope 2.0 were utilized for B-cell epitope prediction. PyMOL was used for protein structural analysis.

**Results:**

36 enriched peptides were identified by biopanning with antibodies from two COVID-19 patients; the peptides 4 motifs with consensus residues corresponding to two potential B-cell epitopes on SARS-CoV-2 viral proteins. The putative epitopes and hit peptides were then synthesized for validation by competitive antibody binding and serological detection.

**Conclusions:**

The identified B-cell epitopes on SARS-CoV-2 may aid investigations into COVID-19 pathogenesis and facilitate the development of epitope-based serological diagnostics and vaccines.

## Background

With the occurrences of severe acute respiratory syndrome (SARS) in 2003 and Middle East respiratory syndrome (MERS) in 2012, infectious disease pandemics have become an increasingly common threat to global health. Since it was first reported in November 2019, coronavirus disease-19 (COVID-19) has spread from Wuhan, China to six continents and over 180 countries (as of June 30, 2020). As 7 March 2021, 116,822,839 people around the world have been diagnosed, and 2,593,073 deaths from COVID-19 have been recorded (case fatality rate: 2.22%, John Hopkins Coronavirus Resource Center, accessed 7 March 2021) [1]. COVID-19 has a highly variable clinical presentation, and in the most serious cases, it is characterized by fever, severe acute respiratory syndrome with pneumonia, and diffuse alveolar damage. Social distancing and city-wide lockdowns have been utilized to efficiently decrease the virus spread, but these measures may also cause serious economic harm during disease outbreaks. It is therefore urgent to develop diagnostic tools and therapeutic strategies for COVID-19.

The virus causing COVID-19 is called severe acute respiratory syndrome coronavirus 2 (SARS-CoV-2) [2, 3]. It is in the *Betacoronavirus* genus and was named based on its phylogenetic classification as a severe acute respiratory syndrome-related coronavirus species [2, 4]. Like SARS-CoV and MERS-CoV, SARS-CoV-2 is subject to cross-species transmission. It has a genome size of approximately 30 kb and encodes 16 orf1ab non-structural proteins, four structural proteins (nucleocapsid, spike, envelope and membrane) and at least six accessory proteins [5]. Since SARS-CoV-2 is a recently discovered pathogen, immunological information about antibody-eliciting epitopes and T cell response is still lacking. Discovery of epitopes, especially for relatively uncharacterized pathogens, is a pivotal step in understanding the pathogenesis of viral infections and in developing diagnostic reagents and epitope-based vaccines, as demonstrated for Hantaviruses [6]. A recent study used homologous SARS-CoV sequences to predict potential B-cell and T-cell epitopes on SARS-CoV-2 viral proteins [7]. However, another study showed that many SARS-CoV monoclonal antibodies recognizing the immunologically important spike receptor binding domain (RBD) cannot recognize the SARS-CoV-2 spike RBD; this lack of recognition is due to low sequence homology in the RBD region, especially within the critical receptor binding motif (RBM) [8]. Hence, there is an unmet need to identify immunologic SARS-CoV-2 epitopes using other more direct approaches.

Phage-displayed peptide libraries have been developed and successfully utilized to identify immunogenic targets or epitopes on viruses, such as those causing dengue fever [9]. In addition, phage-displayed random 12-mer peptide libraries were used successfully to discover specific B-cell epitopes for SARS-CoV; IgG/IgM-combined serological detection of these epitopes showed 100% sensitivity in seven patients and 100% specificity in 22 healthy control subjects [10]. Thus, the phage-displayed peptide library is a powerful platform to rapidly identify disease-specific B-cell epitopes that may provide a foundation for diagnostic tools and epitope-based vaccine design.

In this study, we collected serum samples from COVID-19 convalescent patients and heathy individuals to screen for COVID-19-specific B-cell epitopes. Ultimately, eight potential B-cell epitopes were identified and prioritized using a public epitope-database and viral protein antigenicity prediction. We then performed validation studies on synthesized hit peptides and putative SARS-CoV-2-specific B-cell epitopes. This information is expected to aid in investigations into COVID-19 pathogenesis and the development of laboratory and clinical diagnostic tests.

## Materials and methods

### Human serum samples

Twenty-five COVID-19 patients with illness meeting the CDC definition of probable COVID-19 and presenting positive results on PCR-based confirmation diagnostics were recruited. Patients were considered convalescent when they achieved negative results in three independent PCR-based tests. Serum specimens from the 25 convalescent patients were sent to the Department of Laboratory Medicine for routine serologic and biochemical analyses. The serum samples were collected and handled in accordance with ethical and institutional guidelines and with the approval of the Institutional Review Board at Academia Sinica (AS-IRB02-109044 and AS-IRB02-109158).

### Affinity selection of phages by biopanning

Protein G magnetic beads (#1003D, Thermo Fisher Scientific) were used to capture IgG from the sera of COVID-19 patients and the mixture of eight sera from normal healthy donors (NHS). Briefly, protein G beads were blocked with 1% BSA overnight, followed by incubation with serum samples for 80 min at room temperature. A total of 2 × 10^11^ plaque forming units (pfu) of UV-inactivated M13KO7 Helper Phage (N0315S, New England BioLabs) was used to block IgG-captured beads. After 30 min blocking at room temperature, 1 × 10^11^ pfu phage-displayed 12-mer peptide library (E8111L, New England BioLabs) was incubated with NHS-captured beads for 50 min at room temperature. Next, the phages that remained unbound to NHS-captured beads were incubated with COVID-19 patient sera-captured beads for affinity selection for 1 h at room temperature. Magnetic beads were washed extensively with PBS containing 0.1% Tween-20 (PBST_0.1_) six times to remove unbound phages. Bound phages were eluted with 0.2 M glycine buffer (pH 2.2), neutralized with 1 M Tris buffer (pH 9.1), and amplified for subsequent rounds of selection. Three rounds of selection were performed for each patient. The biopanning protocol for the second and third rounds was identical to that of the first round, with the addition of 2 × 10^11^ pfu of amplified phages for biopanning. Phage titration was performed on Luria broth/isopropyl b-d-thiogalactoside/5-bromo-4-chloro-3-indolyl-b-d-galactoside plates (Falcon; Becton Dickinson). Individual phage clones from the third round were further selected for ELISA screening using COVID-19 patient sera. DNA from immunopositive phage clones was extracted by iodide buffer (10 mM Tris–HCl, pH 8.0, 1 mM EDTA, 4 M sodium iodide) for DNA sequencing using the primer: 5ʹ-CCC TCA TAG TTA GCG TAA CG-3ʹ. The sequences of phage-displayed peptides were aligned to the SARS-CoV-2 protein sequences derived from the NCBI reference genome (Accession number: NC_045512).

### B-cell epitope prediction

The accession identification numbers of 11 SARS-CoV-2 proteins are: ORF1ab polyprotein (NCBI: YP_009724389.1), spike glycoprotein (S) (NCBI: YP_009724390.1), ORF3a (NCBI: YP_009724391.1), envelope protein (E) (NCBI: YP_009724392.1), membrane glycoprotein (M) (NCBI: YP_009724393.1), ORF6 (NCBI: YP_009724394.1), ORF7a (NCBI: YP_009724395.1), ORF7b (NCBI: YP_009725318.1), ORF8 (NCBI: YP_009724396.1), NP phosphoprotein (N) (NCBI: YP_009724397.2), ORF10 (NCBI: YP_009725255.1). Immune Epitope Database and Analysis Resource (IEDB) B Cell Prediction Tools were used to predict epitopes on different SARS-CoV-2 viral proteins. Linear B-cell epitope prediction was performed using the BepiPred-2.0 [11] algorithm with cutoff value of 0.55 (specificity greater than 0.8). Prediction of structure-based epitopes from viral proteins was conducted with the DiscoTope 2.0 algorithm using SARS-CoV-2 spike (PDB: 6VSB) and nucleocapsid (PDB: 6M3M and 6WJI) structures, and cutoff of − 2.5 with specificity equal to 0.8 [12]. Epitope residues predicted by linear- and structure-based methods were further validated with VaxiJen, which predicts antigenicity based on the physicochemical properties of proteins; VaxiJen is alignment-independent, and shows accuracy of 70% to 89% [13]. The cutoff of 0.4 for prediction of viral antigens was used, as suggested by VaxiJen. Finally, sequences with conserved residues of phage-displayed peptides, BepiPred-2.0 score above 0.55, DiscoTope 2.0 score above -2.5 and VaxiJen score above 0.4 were considered potential COVID-19 B-cell epitopes.

### Serological detection by enzyme-linked immunosorbent assay (ELISA)

For phage testing, an ELISA plate (#442404, Thermo Fisher Scientific Inc.) was coated with 0.5 μg/ml purified anti-human IgG antibodies (#109-035-088, Jackson ImmunoResearch Labs) in 0.1 M NaHCO_3_ (pH 8.6) for 6 h at 4 °C, and was then blocked with PBS containing 1% bovine serum albumin (BSA) at 4 °C overnight. The IgG-coated plates were incubated with the sera of COVID-19 patients (1:150 dilution) at room temperature for 1 h. Then, the plates were washed three times with PBST_0.1_. Selected and diluted phage clones (5 × 10^8^ or 1 × 10^9^ pfu per well) were incubated with COVID-19 patient antibody-coated plates for 1 h. After six washes with PBS containing 0.5% Tween-20 (PBST_0.5_), HRP-conjugated anti-M13 antibody (#27-9421-01, GE Healthcare) was prepared in PBS containing 1% BSA (1:5000 dilution) and added into plate for another 1-h incubation. Plates were washed six times with PBST_0.5_ followed by TMB color development (TMBW-1000-01, SURMODICS™). The reaction was stopped with 3 N HCl, and absorbance was measured at 450 nm by ELISA reader (Versa Max Tunable Microplate Reader; Molecular Devices).

For serological detection of recombinant proteins and synthetic peptides, ELISA plates were coated with 50 μl/well of recombinant protein (0.5 μg/ml) or synthetic peptides (10 μg/ml) for 6 h at 4 °C, followed by 3% skin milk (in PBS) blocking at 4 °C overnight. COVID-19 patient sera were diluted in milk blocking buffer (1:250) and added into coated plate for 1-h incubation at RT. After three washes with PBST_0.1_, the plate was incubated with HRP-donkey anti-hIgG (1:10,000 dilution in milk blocking buffer) (#709-035-149, Jackson ImmunoResearch Inc.) for 1 h at room temperature, followed by TMB color development.

### Antibody competitive inhibition assay

An ELISA plate was coated with anti-human IgG antibodies to capture COVID-19 serum IgG. Serially diluted synthetic peptides were mixed with 1 × 10^9^ pfu phages, and the peptide/phage mixtures were added to a COVID-19 IgG-coated plate for 1-h incubation. After six washes with PBST_0.1_, the plate was incubated with HRP-conjugated anti-M13 antibody (1:5000 in 1% BSA) for 1 h followed by ELISA measurement, as described above.

### Statistical analysis

Data are presented as mean ± SD. The cutoff for serologic detection was calculated as [mean of OD_450_ + 4 × SD] of normal healthy serum samples.

## Results

### Identification of SARS-CoV-2-specific B-cell epitopes with phage-displayed peptide libraries

**Fig. 1:**
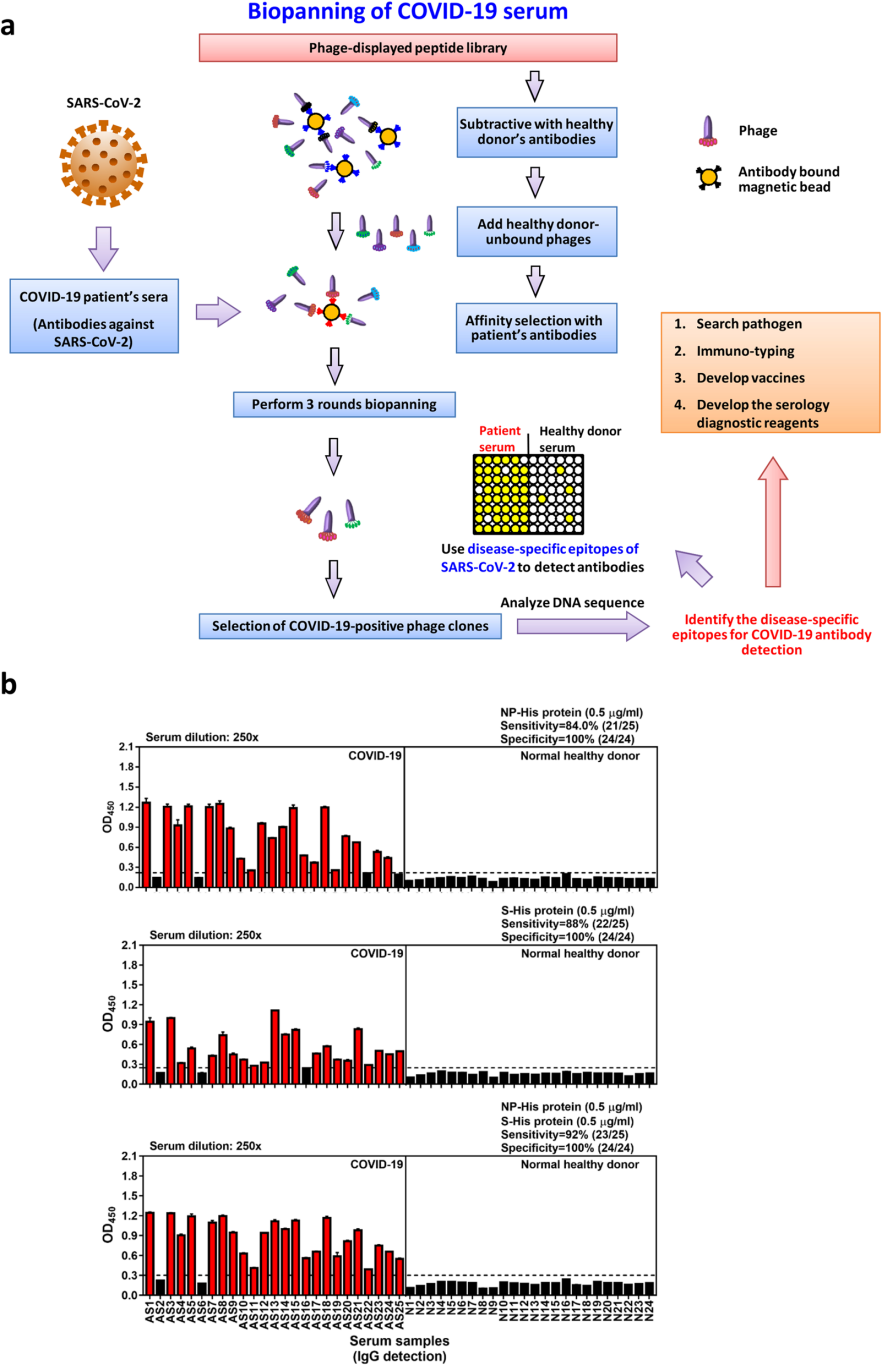
Biopanning phage-displayed peptide library with COVID-19 patient serum samples. **a** Illustration shows the strategy for biopanning disease-specific B-cell epitopes. Serum samples of COVID-19 patients and normal healthy donors were used to prepare IgG-captured magnetic beads. The phage-displayed peptide library was pre-cleaned by normal healthy serum (NHS) IgG-coated magnetic beads, and three rounds of affinity selection of NHS-unbound phages were performed using COVID-19 IgG-magnetic beads. After biopanning, immunopositive phage clones were validated with ELISA and DNA sequenced. The disease-specific epitopes were further identified and characterized by bioinformatic prediction of B-cell epitopes, structural analysis and SARS-CoV-2 protein/peptide synthesis for binding and competitive-inhibition assays. Information about disease-specifc epitopes will be helpful for pathogen research, immune-typing, development of vaccines and serology diagnostic reagents. **b** Serological detection of COVID-19 convalescent patient antibodies using SARS-CoV-2 NP and extracellular domain of spike recombinant proteins. Horizontal dashed line denotes the cutoff value (calculated as mean OD_450_ + 4 × SD of normal healthy serum samples) for recombinant SARS-CoV-2 NP or extracellular domain of S protein. Immunopositive cases are colored red

To find SARS-CoV-2-specific B-cell epitopes, we used phage display of 12-mer random peptide libraries to screen serum samples from COVID-19 convalescent patients (Fig. 1a). Sera from normal healthy donors (NHS) was used in a pre-screen to eliminate common human IgG-bound phages, and the pre-cleaned phage libraries were panned with COVID-19-serum-IgG-conjugated beads. After three rounds of biopanning, individual phage clones were validated by ELISA screening, and the DNA sequences of immunopositive clones were determined, followed by alignment analysis and B-cell epitope prediction.

**Fig. 2:**
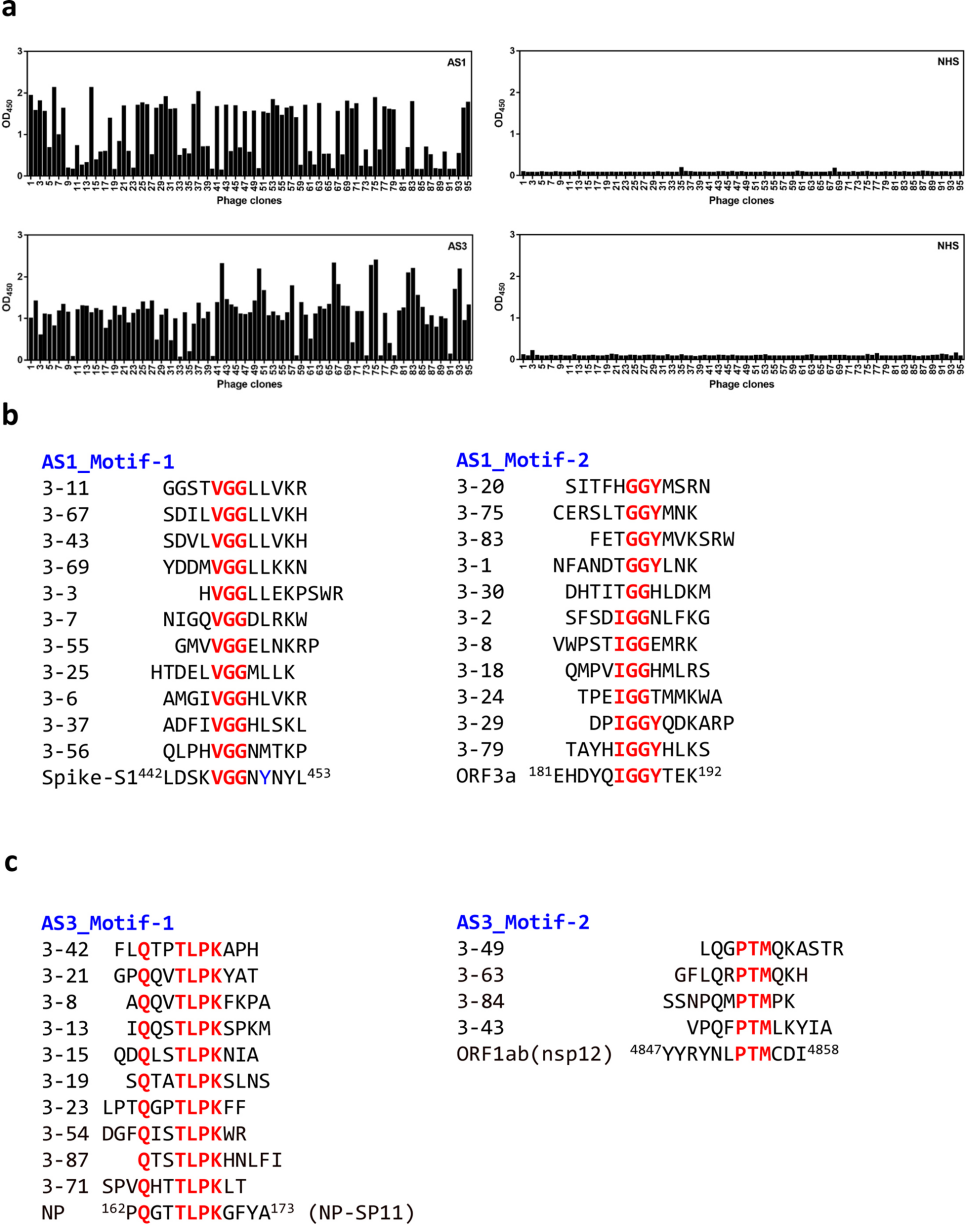
Identified peptides from COVID19 positive reacted phage clones. **a** A phage-displayed random peptide library was screened with serum antibodies from patient AS1 and AS3, respectively. After three screening rounds, enriched phage clones were significantly reactive to antibodies in serum samples from COVID-19 patient but not to samples of normal human serum (NHS). **b**, **c** Alignment analysis of sequenced peptides from AS1 (**b**) and AS3 (**c**) biopanning was conducted, and identified the conserved residues corresponding to SARS-CoV-2 viral proteins

We collected serum samples from 25 convalescent COVID-19 patients and 24 normal healthy donors. Then, we performed serological testing using recombinant nucleocapsid phosphoprotein (NP) and extracellular domain of spike (S) glycoprotein as antigens, in order to validate disease-specific immunity in the COVID-19 patients. In our serological testing, NP protein was recognized by 21 of 25 COVID-19 sera (sensitivity: 84%), and S was bound by 22 of 25 COVID-19 sera (sensitivity: 88%) (Fig. 1b). The use of both NP and S increased the detection sensitivity to 92%, and the specificity was 100%. Notably, the patient serum samples, AS2 and AS6, showed negative reactivity with both NP and S proteins (Fig. 1b). Based on the serological testing data, two positive cases (AS1 and AS3) were selected for biopanning of phage-displayed peptides (Fig. 2a). After sequencing and alignment analysis of positive phage clones, highly conserved residues corresponding to different SARS-CoV-2 viral protein sequences were further assessed for B-cell epitope and antigenicity prediction (Fig. 2b, c). The potential SARS-CoV-2 B-cell epitopes identified from biopanning with COVID-19 sera are shown in Table 1.

**Table 1:**
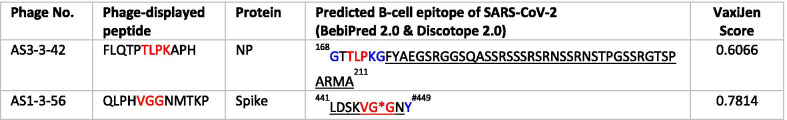
Phage-displayed peptides recognized by COVID-19 convalescent patient IgG

Conserved amino acids in putative epitope residues are colored red

Linear B-cell epitope is denoted by bottom line, while structure B-cell epitope is colored blue

Sequences from predicted B cell epitopes with VaxiJen Score above 0.4 were considered potential COVID-19 B-cell epitopes

^a^Residues are reported to involve in Spike RBD/ACE2 interaction [18]

### Validation of putative B-cell epitopes on NP

**Fig. 3:**
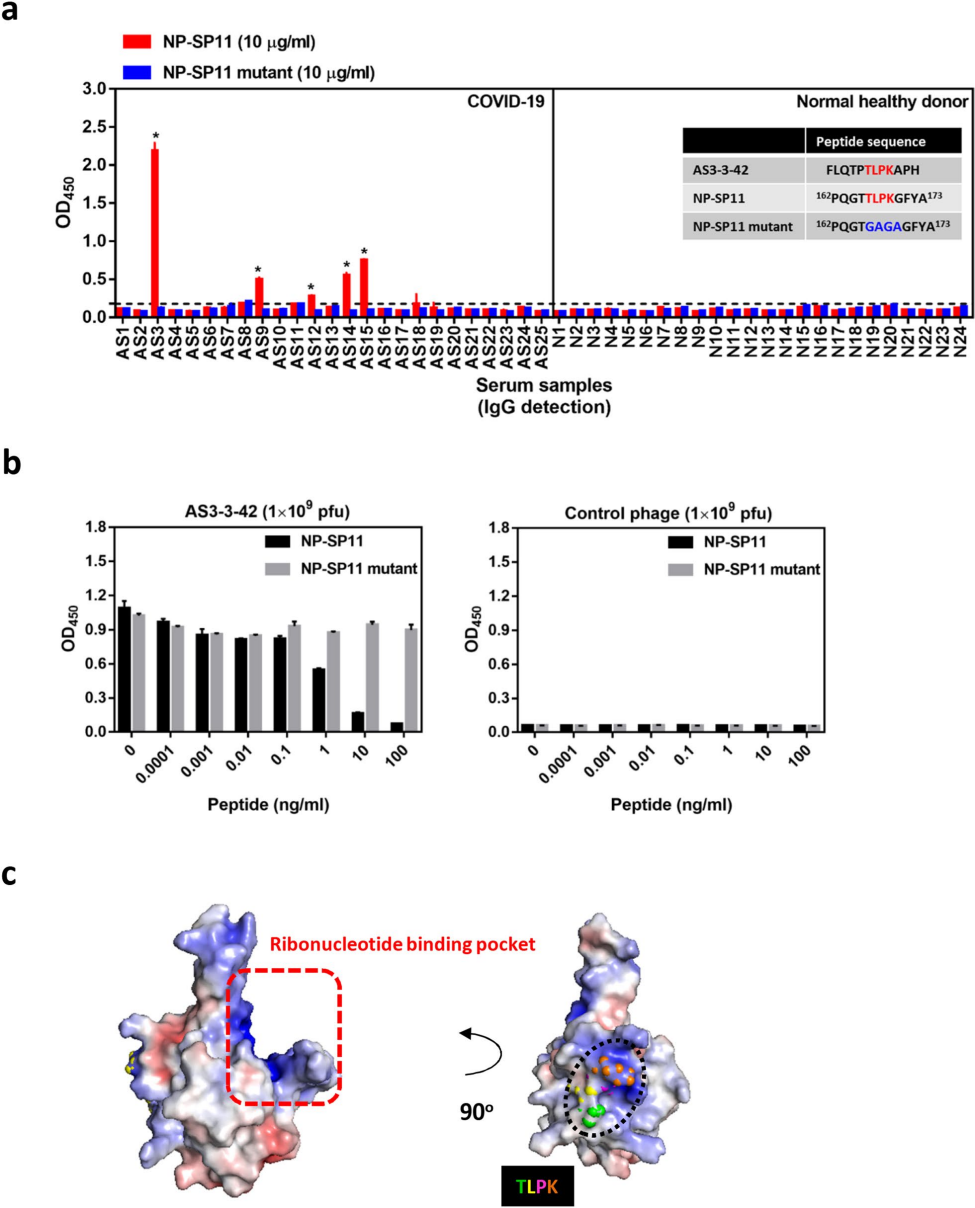
Validation of COVID-19 disease-specific B-cell epitopes derived from biopanning of phage-displayed random peptide libraries. **a** Serological detection of COVID-19 convalescent patients using NP-SP11 and NP-SP11 mutant peptides. Peptide (2.5 μg/well; 10 μg/ml) was coated overnight followed by serum incubation. Patient IgG binding to peptide was detected by anti-human IgG HRP secondary antibody, and the OD was measured at 450 nm. Sequence of AS3-3-42 phage-displayed peptide and synthetic SARS-CoV-2 NP protein peptides with TLPK motif (NP-SP11) or the GAGA-mutated motif (NP-SP11 mutant) are shown. **b** Peptide competition assay. IgG from AS3 patient serum was captured, followed by 1-h incubation with both 1 × 10^9^ pfu AS3-3-42 phage (or control phage) and different amounts of synthetic NP peptide. Phage binding was detected by anti-M13 HRP secondary antibody and OD was measured at 450 nm. **c** Electrostatic surface of the SARS-CoV-2 NP N-terminal domain (NP-NTD, PDB ID: 6M3M). The potential charge distribution was calculated by PyMOL. Blue color indicates positive charge potential; red color indicates negative charge potential. Dashed line denotes the potential ribonucleotide binding pocket [14]. TLPK residues are shown as a sphere. Horizontal dashed line denotes the cutoff value (calculated as mean OD_450_ + 4 × SD of normal healthy serum samples) for AS3-3-42 phage or NP-SP11 peptide. * Indicates COVID-19-specific positive case

Next, we sought to validate the putative B-cell epitopes by determining whether the epitopes could be used to detect antibodies in COVID-19 patient serum samples. Here, the corresponding NP-SP11 synthetic peptide reacted with five of the seven patient serum samples (AS3, AS9, AS12, AS14, AS15) (Fig. 3a). To further determine whether the conserved TLPK residues are important for peptide recognition by COVID-19 patient sera, we changed the TLPK residues to GAGA (NP-SP11 mutant). Serum testing data showed that the NP-SP11 mutant peptide completely lacked reactivity with all COVID-19 patient serum samples (Fig. 3a). Notably, competitive binding experiments revealed that the NP-SP11 mutant peptide cannot compete with AS3-3-42 phage binding to COVID-19 serum antibodies (Fig. 3b). Thus, our validation data reveal a requirement for TLPK residues in the antigen for COVID-19 antibody recognition.

Since the TLPK-containing B-cell epitope is located within the N-terminal domain of NP, we next analyzed the crystal structure of the NP N-terminal domain (PDB ID: 6M3M). PyMOL analysis revealed the surface electrostatic potential characteristics of SARS-CoV-2 NP protein; a potential ribonucleotide binding pocket in the positively charged groove of the β-sheet core is denoted by the red dashed line in Fig. 3c (left panel) [14]. After a leftward 90° rotation, the TLPK residues can be seen as spheres on the surface (Fig. 3c, right panel), revealing the spatial arrangement of critical residues recognized by COVID-19 patient antibodies.

### Epitopes in SARS-CoV-2 spike glycoprotein

**Fig. 4:**
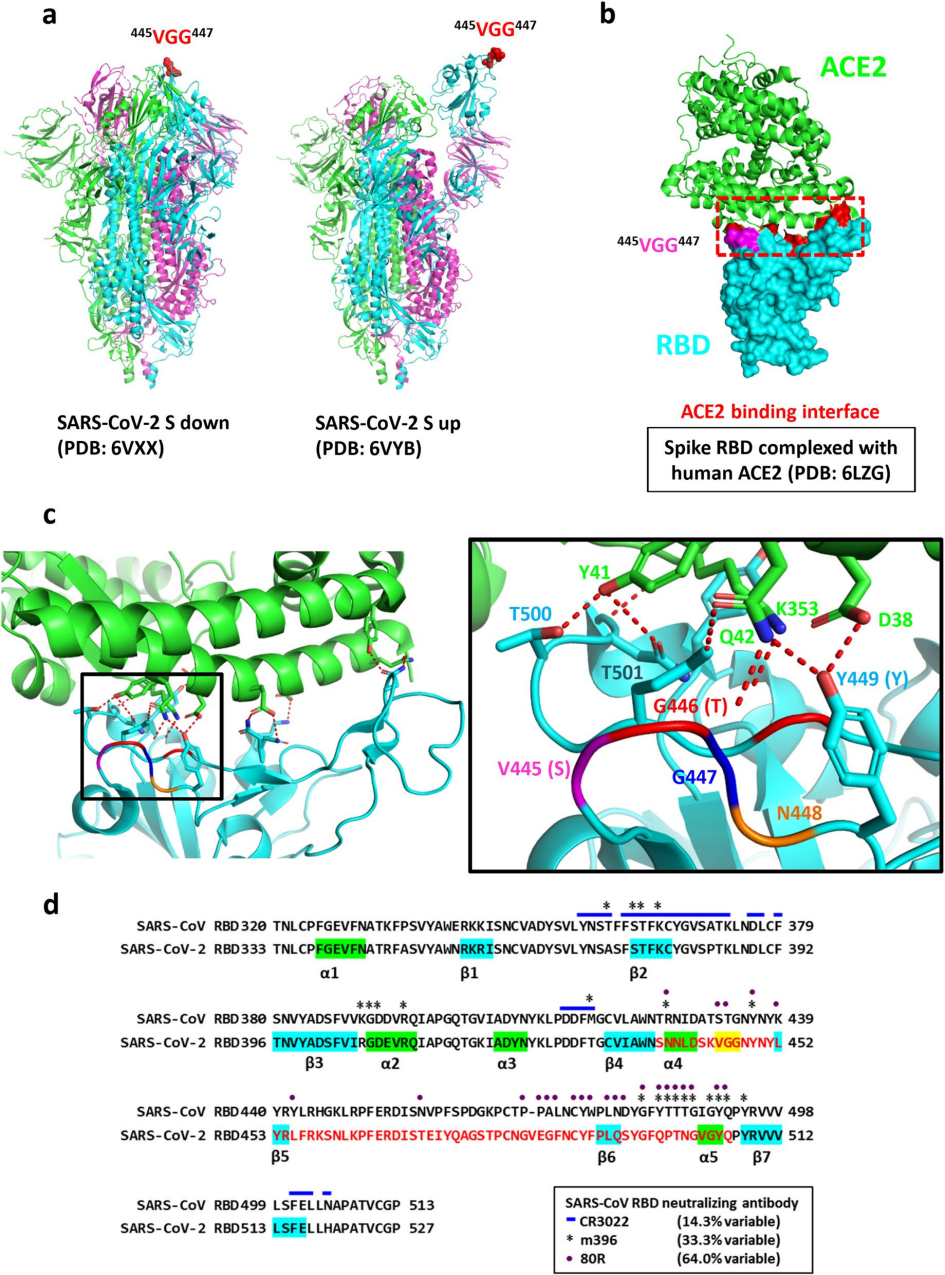
Location of VGG residues in SARS-CoV-2 spike glycoprotein. **a** Conserved VGG residues in the SARS-CoV-2 spike protein are shown as spheres in the prefusion conformation. **b** VGG residues in SARS-CoV-2 spike RBD are colored pink. ACE2 receptor is shown as a ribbon structure and colored green, while SARS-CoV-2-RBD is shown in the surface representation and colored cyan. RBD residues involved in receptor interaction [18] are colored red, and the ACE2 binding interface is marked by a red dashed outline. **c** Details of the polar contacts between SARS-CoV-2-RBD and ACE2 are indicated. Polar interactions are denoted as red dashed lines. Side chains of residues are shown as sticks (left panel). The position of VGG residues corresponds to SARS-CoV RBD serine (S), threonine (T) and glycine (G) (right panel). **d** Comparison of SARS-CoV and SARS-CoV-2 RBD sequence. The reported epitope residues of CR3022, m396 and 80R SARS-CoV neutralizing antibodies are denoted by blue lines, asterisks and purple points, respectively [18, 24]. VGG conserved residues derived from COVID-19 sera/phage-displayed peptide library biopanning are colored yellow

In addition to the consensus TLPK residues in SARS-CoV-2N protein, we also found the other COVID-19 sera-bound peptide containing conserved residues. The ^445^VGG^447^ residues are present within the RBM region (438–506) of the S1 subunit. Since the ‘up’ conformation of the spike RBD has been reported to be the ACE2 bound form, the prefusion up (PDB: 6VYB) and down (PDB: 6VXX) conformations of S were used for structural analysis. The locations of VGG residues in the prefusion conformation of the S protein are shown in the surface of trimeric spike complex (Fig. 4a). In addition, structural analysis of the RBD complexed with human ACE2 receptor (PDB: 6LZG) showed that the VGG residues are in close proximity to the RBD/ACE2 binding interface (Fig. 4b). Interestingly, previous study has reported that the first G (G446) in the VGG sequence forms a hydrogen bond with Q42 of ACE2; [15]. As compared to SARS-CoV sequence, the VGG corresponding residues in SARS-CoV RBD are S432 and T433 (Fig. 4c). This observation raises the possibility that antibody recognition of the epitope may contribute to neutralization of SARS-CoV and/or SARS-CoV-2. Indeed, the corresponding ST residues in SARS-CoV have been reported to be the epitope for SARS-CoV 80R neutralizing antibody (Fig. 4d).

Unfortunately, the SARS-CoV-2 RBM sequence only shares 48% identity with SARS-CoV; hence, SARS-CoV neutralizing antibodies targeting the RBM epitope are unlikely to bind the SARS-CoV-2 RBD. The epitope residues of m396 and 80R neutralizing antibodies are highly diverged compared to those of the CR3022 clone. Recent studies have shown that m396 and 80R do not exhibit binding to the SARS-CoV-2 RBD, due to the high sequence divergence in the RBM [8, 15]. Hence, the identification of epitopes from convalescent COVID-19 patients, such as the VGG at the receptor binding interface, may be especially helpful in the development of vaccines specifically targeting SARS-CoV-2. These epitopes may also be useful for detecting whether infected patients have begun to generate neutralizing antibodies.

## Discussion

Identification of viral B-cell epitopes is important for understanding of virus-antibody interactions at a molecular level, which is crucial information for the development of virus-specific serologic diagnostic reagents and subunit vaccines. This is the first study to characterize serotype-specific B-cell epitopes of SARS-CoV-2 with phage display. By using serum samples of COVID-19 for biopanning, we were able to identify conserved residues that correspond to SARS-CoV-2 viral proteins (Fig. 2b, c), leading to the identification of two putative B-cell epitopes on the viral proteins, NP and S (Table 1).

The SARS-CoV-2 NP protein sequence is 90% identical to that of SARS-CoV, and three dominant B-cell epitope regions (42–62, 153–172 and 355–401) were predicted for the SARS-CoV-2 NP protein using the homologous sequence analysis and the IEDB (Immune Epitope Database and Analysis Resource) [7]. Among these three regions, the corresponding 154–175 region in SARS-CoV NP showed high immunoreactivity in SARS-CoV patient serum samples, and it was strongly immunogenic in mice, macaques and humans [16]. Here, phage-displayed peptides derived from biopanning with AS3 patient serum contained highly conserved TLPK residues (Fig. 2c). A peptide competition assay validated the antigenicity of this putative NP epitope, and a residue mutation assay further demonstrated the importance of TLPK motif for recognition by COVID-19 sera antibodies (Fig. 3a, b).

Because phage-displayed peptides with VGG consensus residues corresponding to residues 445–447 of the spike RBM were enriched by biopanning with COVID-19 sera (Fig. 2b), we expected that the loop region containing these residues may function as a dominant epitope in S for SARS-CoV-2 neutralizing antibodies. Surprisingly, a recent report showed that the 444, 446–452 amino acids (KXGGNYNXL) of spike RBM are the epitope for SARS-CoV-2 neutralizing antibody P2B-2F6 [17], which shows very high RBD binding affinity and strong neutralizing activity against live SARS-CoV-2 infection. Notably, a competitive binding assay showed that the epitopes of six human neutralizing antibodies may be highly overlapping. Moreover, structural analysis showed that G446 forms a hydrogen bond with Q42 of the human ACE2 receptor [18], and G447 forms a hydrogen bond with Y27 of the P2B-2F6 neutralizing antibody heavy chain [17]. Hence, this report of a human neutralizing antibody against SARS-CoV-2, and several structural analyses lead us to strongly suspect that the VGG containing epitope (441–449) between α4 helix and β5 sheet may play an important role in the generation of neutralizing antibodies. Furthermore, this site has high potential for use in tools to assess whether convalescent patients have VGG-targeting neutralizing antibodies. In addition, the VaxiJen prediction tool yielded a high protective antigen score for this identified B-cell epitope (Table 1), further strengthening the idea that the VGG-containing epitope has high potential for development of epitope-based vaccines.

A possible reason for the low number of identified B-cell epitopes is that the peptide-displayed phage library we used might have low diversity. It may therefore be useful to establish a phage-displayed peptide library with higher diversity and use more serum samples from COVID-19 patients to increase the number of identified B-cell epitopes. Alternatively, it is possible that some epitopes might be dominantly recognized by the IgGs in COVID-19 patients, which would mean that only those dominant epitopes are likely to be identified by this method. In addition, non-neutralizing B-cell epitopes are often immunodominant and can be useful as diagnostic targets, while neutralizing epitopes are more rarely targeted and frequently not easily exposed. Hence, the identified B-cell epitopes of COVID-19 from phage-displayed peptide library will require further characterization.

Importantly, a recent study showed that an antibody isolated from 10 convalescent patients potently neutralizes SARS-CoV-2 by binding to the N-terminal domain, but it does not bind to the RBD in spike protein [19]. This report suggested that SARS-CoV-2 neutralizing antibodies do not only bind to epitopes in RBD domain, and the phage-displayed peptide library used for epitope screening of antibodies from convalescent patients may be useful to discover RBD-binding and non-RBD-binding epitopes.

Interestingly, the SARS-CoV-2 S epitope containing VGG that we identified in this study were also predicted to be a potential T-cell epitope in a bioinformatic analysis [7]. Moreover, the VGG-containing region was shown to be a T cell epitope in INO-4800-immunized BALB/c mice [20]. Several single-cell sequencing studies have reported inflammatory storms in COVID-19 patients, with high proinflammatory monocytes/macrophages accompanied by decreased T cells [21]. In addition, moderate COVID-19 cases are characterized by the presence of highly clonally expanded CD8^+^ T cells (e.g. ZNF683^+^ CD8 T cells) in contrast to severe patients [22]. These observations strongly suggest that T cell-elicited immunity is tightly associated with clinical outcome and may be beneficial for the recovery of COVID-19 patients [22]. The B-cell epitopes of SARS-CoV-2 S protein identified in this study and predicted as potential T-cell epitopes (e.g. VGG-containing epitopes) will be an attractive target for the development of a powerful epitope-based vaccine that can elicit anti-viral innate and humoral immunity against SARS-CoV-2 infection.

The current method of diagnosing COVID-19 involves collecting respiratory tract samples (sputum, lower respiratory tract aspirate or throat swab), feces or blood of patients suspected of being infected with SARS-CoV-2. Nucleic acid fragments extracted from these samples are then subjected to fluorescent RT-PCR in order to positively identify pathogenic strains by genomic sequence. If the SARS-CoV-2 nucleic acid sequence is positively identified, the patient is confirmed as infected. This method of diagnosis is currently used in all countries to confirm COVID-19 patients. However, there are several disadvantages to this approach. First, in order to conduct PCR, the samples must be pre-processed, and specialized instruments are required. Without correct sample processing, a high false-negative rate or low sensitivity will result. Additionally, the whole procedure takes 4 h to complete, and it must be conducted in specialized laboratories operating under stringent safety guidelines. Not all hospitals have such specialized laboratories or appropriately trained personnel to perform the diagnostic test, so the capacity of carrying out diagnostic testing on a large scale is often limited.

Another diagnostic method is antigen detection, wherein an antibody is used to probe for the presence of SARS-CoV-2 antigen. This antigen–antibody reaction method typically has high specificity. It is also the easiest and fastest detection method available at present. The only required material is paper, and results can be obtained in 15–20 min. This simple and fast method of detection is suitable for large-scale screening and therefore represents an ideal first-line defense against massive viral contagion outbreak in large populations. It is worth noting, however, that the antigen test has a window period during which it cannot reliably identify infected patients. This window occurs just after the patient has been infected, when the amount of virus is still extremely low. In such cases, the viral antigen is below the limit of detection, and the test is prone to false negatives. Therefore, for patients with suspected COVID-19, the nucleic acid detection method should be used in conjunction with the antibody-antigen test to increase the accuracy of screening results.

A third detection method involves probing for SARS-CoV-2 antibody in the patient’s serum (Fig. 1b). This method uses viral antigens to detect the presence of circulating antibodies against the virus. Because antibodies will appear in patients only after they contract the disease, this detection method is suitable for detecting infection at the middle or later stages of disease progression. For this reason, antibody tests are mostly used as an auxiliary detection method to facilitate diagnosis. In this study, we showed that the sensitivity of anti-NP (84%) is slightly lower than anti-S (88%). This observation may be correlated with the age of patients. In clinical cases, SARS-CoV-2 infection in adults and children leads to different antibody responses. Anti-NP IgG is not usually prevalent in pediatric patients with or without multisystem inflammatory syndrome (MIS), but anti-S IgG is abundant. However, in adult convalescent plasma donors (CPDs), ELISA detection shows high levels of both anti-S and anti-NP IgG antibodies [23]. Dual detection of anti-NP and anti-S using NP and S protein can increase the sensitivity of serum antibody detection (Fig. 1b).

The B-cell epitopes we identified by biopanning a phage display library may be useful to generate epitope-based diagnostic reagents (Fig. 2). Our data suggest that an epitope-based diagnostic reagent would be able to detect SARS-CoV-2 antibodies with high specificity, as the antigens we identified did not cross-react with control serum samples. Furthermore, we found that using NP as a target to detect COVID-19 could lead to false-negative results. In our clinical samples, two patients (AS2 and AS6) out of 25 showed false-negative results (Fig. 1b). This limitation might be overcome by the use of other or multiple epitope-based diagnostic reagents.

With well-characterized pathogens, molecular biology approaches may be used to rapidly generate antigens for antibody detection in infected patients. The manufacture of such antigens can be accomplished with relative ease and low cost. Therefore, the use of antigens as a tool for diagnostic reagents has the advantages of speed, ease of use and low cost. However, when patients are asymptomatic and in early stages of infection, this diagnostic approach does not work well. In addition, if the pathogen has a large number of close relatives with a high degree of similarity, this diagnostic approach might also work poorly. In such cases, the antigen might not be able to accurately distinguish the target pathogen from other similar pathogens, potentially leading to high rates of false positives. There are seven known types of human coronaviruses that can lead to respiratory infections, which range from the common cold to severe diseases like MERS, SARS or COVID-19. Therefore, the use of antigen detection for SARS-CoV-2 might suffer from high rates of false-positive results.

It is likely that many people have already been exposed to and developed antibodies toward the four types of coronavirus that do not usually lead to severe symptoms. Thus, our use of serum to detect SARS-CoV-2 epitopes could have easily led to false-positive results. To avoid this obstacle, we used control sera from uninfected patients to pre-clean the library before probing with sera from convalescent COVID-19 patients. In this way, we were able to improve our chance of isolating epitopes specific to SARS-CoV-2. We also performed analyses to identify the corresponding viral proteins and actual epitopes detected by serum from infected patients. These specific epitopes can be used for the future development of neutralizing antibodies or vaccines.

In summary, we identified B-cell epitopes for SARS-CoV-2 using a phage-displayed peptide library and COVID-19 sera biopanning. Our results provide detailed information about pathogen epitopes that can help in the rapid development of immunodiagnostic tools for first-line detection of disease outbreak and also show promise for guiding epitope-based vaccine design for disease control.

## Conclusions

To rapidly identify the disease-specific B-cell epitopes that correspond to or mimic natural epitopes, we used phage display technology to determine the targets of specific antibodies present in the sera of COVID-19 patients. In this study, we identified the enriched peptides from the sera of COVID-19 patients using biopanning, and classified the peptides with consensus residues corresponding to potential B-cell epitopes on SARS-CoV-2 viral proteins. The putative epitopes and hit peptides were then synthesized for validation by competitive antibody binding and serological detection. Taken together, these B-cell epitopes on SARS-CoV-2 may aid investigations into COVID-19 pathogenesis and facilitate the development of epitope-based serological diagnostics and vaccines.

## Data Availability

All materials are available by the corresponding author.

## Abbreviations

COVID-19: Coronavirus disease 2019
SARS-CoV-2: Severe acute respiratory syndrome coronavirus 2
S: Spike
NP: Nucleocapsid phosphoprotein
RBD: Receptor binding domain
RBM: Receptor binding motif
NHS: Sera from normal healthy donors
pfu: Plaque forming units
